# Conditioned Place Preference Induced by Licit Drugs: Establishment, Extinction, and Reinstatement

**DOI:** 10.1100/tsw.2008.154

**Published:** 2008-12-14

**Authors:** Yu Liu, Bernard Le Foll, Yanli Liu, Xi Wang, Lin Lu

**Affiliations:** ^2^ Centre for Addiction and Mental Health and University of Toronto, Canada; ^1^ National Institute on Drug Dependence, Peking University, Beijing, China; ^3^ College of Pharmacy, Soochow University, Suzhou, China

**Keywords:** conditioned place preference, rewarding effect, ethanol, nicotine, caffeine

## Abstract

The conditioned place preference (CPP) model has been widely used to evaluate the rewarding effects of abused drugs, and recently, the extinction and reinstatement phases of this paradigm have been used to assess relapse to drug seeking. The vast majority of studies have focused on CPP induced by illicit drugs, such as psychostimulants and opioids. Although legal psychoactive drugs, such as ethanol, nicotine, and caffeine, are more widely used than illegal drugs, the establishment, extinction, and reinstatement of CPP produced by these licit drugs are less well understood. The present review discusses the extant research on CPP induced by legal drugs. We first describe the CPP model and discuss the behavioral procedures used to induce CPP for ethanol, nicotine, and caffeine. We then summarize the neuronal substrates that underlie CPP induced by these drugs from a genetic perspective. Finally, we draw on findings from pharmacological studies and discuss the neurotransmitters and neurohormones underlying CPP produced by ethanol, nicotine, and caffeine.

### INTRODUCTION

The relatively simple conditioned place preference (CPP) model has been widely used to evaluate the rewarding and aversive effects of drugs. The "precursor" to the current CPP procedure was indeed introduced by Beach in 1957[1]. Interestingly, this study was conducted to examine the drive-reduction hypothesis of addiction learning in rats. Animals with or without additional morphine injections prior to the conditioning session were run in a Y maze with different goal boxes, and both groups later exhibited significant preferences for the morphine-associated goal box. Although this study did not specifically emphasize the rewarding effects of drugs and instrumental responses were involved, it had some conceptual similarities to the current version of place conditioning methods[2]. Kumar modified the procedure by placing animals in the testing chamber immediately after drug injections[3]. Because the animals were maintained on daily morphine injections prior to the CPP task, and high-dose morphine (120 mg/kg) was used during CPP induction, the relief of withdrawal, rather than the rewarding effects of morphine, was

*The authors contributed equally to this paper.
**Corresponding author.

implicated as the main cause of the animals' conditioned responses. In 1976, Rossi and Reid reported for the first time the ability of low-dose morphine to induce a conditioned response in rats successfully. The authors suggested that the drug's "affective consequences" and the positive hedonic impact of drug administration were specifically assessed by CPP testing[4]. The investigation of the rewarding properties of drugs in the past decade has especially prompted the use of the CPP model. The number of studies using the CPP model has rapidly grown, with over 1000 studies published since 1998[5].

Pavlovian conditioning is greatly involved in the establishment of drug-induced CPP. Initially, the drug serves as an unconditioned stimulus (US) and the subjective state produced by the drug is an unconditioned response (UR). During the CPP acquisition phase, the animals receive injections of drugs or saline paired with a distinct environment in the testing apparatus, and develop an association of a specific environment with the subjective effect of the drug. Subsequently, the drug-paired environment becomes a conditioned stimulus (CS) and reaction to the drug becomes a conditioned response (CR). In the following expression (testing) phase, the animals may spend more time in the previously drug-associated environment when the drug is not present in the environment. It should be noted that the CS presumably acts as a conditioned reinforcer and spending more time in the drug-paired environment is therefore an operant response. In general, changes in the days/drug doses for the animals to develop drug-induced CPP and changes in the already established CPP performance are used to measure the effect of a treatment on the acquisition and expression of CPP, respectively.

Similar to learned associations established in other behavioral tasks, CPP can be extinguished and reinstated[6,7,8]. Extinction of previously established drug-induced CPP can be accomplished either by repeated exposure to the preferred environment without drug exposure or by pairing both the previously drug- and nondrug-paired environments with saline (vehicle) injections. The efficiency of CPP extinction depends on several factors, including the number of pairings or injections, the number of extinction trials, and the duration of the extinction period[7,9,60]. Reinstatement of drug-seeking and -taking behavior in the experimental animals is hypothesized to be relevant to drug relapse in humans, and the reinstatement model has been used to identify the behavioral and neural mechanisms underlying relapse[10,11]. Using the CPP procedure, a growing number of studies has also demonstrated that both drug-priming injections and stressors could reinstate previously diminished CPP performance[7,8,12,13,14,15,16]. This phenomenon can be a specific reflection of relapse to drug-seeking behavior that is commonly observed in drug-dependent individuals.

The CPP model has been reviewed in the literature[17,18]. Suzuki[19] provided a comprehensive review on the procedure and related issues. Tzschentke extensively reviewed many key findings from CPP experiments, focusing on the neurobehavioral and neurochemical processes associated with the rewarding effects of drugs[5,20]. Bardo and Bevins also discussed the controversial aspects of the model[21]. These reviews reveal that the addiction field has focused almost exclusively on CPP induced by illicit drugs, such as cocaine, amphetamine, and morphine. However, legal drugs, such as alcohol, nicotine, and caffeine, are more widely used and have significant societal impact[22,23]. Here, we provide the first review of the smaller set of studies on the mechanisms underlying CPP induced by legal drugs. We summarize recent findings in the field and review the behavioral processes, neuronal substrates, and neurochemical mechanisms underlying the establishment (acquisition and expression), extinction, and reinstatement of ethanol-, nicotine-, and caffeine-induced CPP.

### ETHANOL CONDITIONED PLACE PREFERENCE

#### Establishment (Acquisition and Expression) of Ethanol CPP

Voluntary ethanol consumption in animal models using oral self-administration and two-bottle choice procedures has been well documented, and reveals major behavioral differences in the experimental animals depending on the species used and other experimental variables[see reviews, 24,25]. Although researchers have found the demonstration of ethanol CPP in rats to be challenging[26,27,28,29,30,31], robust and reliable CPP has been demonstrated in mice[32,33,34,35,36,37]. In mice, the primary reinforcer ethanol can act as the US. Some studies have demonstrated differences in sensitivity to the rewarding effects of ethanol in rats and mice by subjecting both species to similar CPP paradigms[38]. Notably, the interval between ethanol (US) and environmental exposure (CS) can also be an important determinant of differential CPP performance between rats and mice. For example, studies performed by Cunningham and colleagues demonstrated that CPP (aversion) occurred when ethanol was administered immediately before (after) CS exposure[39]. In addition, a longer interval between ethanol and environmental exposure failed to establish any conditioned response (CPP or CPA), regardless of the order of drug and environmental exposure[40]. Ethanol CPP can also be greatly affected by the number of conditioning trials and dose of ethanol used[41,42,43].

In the past decade, nearly 100 genes have been examined for their potential involvement in ethanol's behavioral effects, including ethanol CPP[44]. For example, dopamine $D_2$ receptor knockout mice show reduced ethanol CPP[45], although CPP remained unaltered in $D_3$ receptor–deficient mice[45,46]. Moreover, reduced ethanol CPP was observed in DARPP-32 (dopamine and adenosine 3,5-monophosphate-regulated phosphoprotein) knockout mice, further suggesting dopamine's role in ethanol CPP[47]. Reduced ethanol CPP has also been found in mice with genetic reduction of many other neuronal substrates, including norepinephrine in the medial prefrontal cortex, vesicular monoamine transporter 2, cannabinoid $CB_1$ receptors, preproenkephalin, preprodynorphin, $GABA_{A\alpha1}$ receptors, $GABA_A$ transporter subtype 1, serotonin 5-$HT_{1B}$ receptors, and the NR2A subunit of the *N*-methyl-D-aspartate (NMDA) receptor[48,49,50,51,52,53,54,55,56]. In contrast, genetic deletion (knockout) of serotonin transporters, μ opioid receptors, endogenous nociceptin receptors, and Fyn kinase has a minimal effect on ethanol CPP[57,58,59,60]. As mentioned previously, learning association provides a basis for the animals to develop drug-induced CPP. Notably, the learned association between environmental stimuli and drug effect provides the basis for the CPP task. It remains unknown to what extent a nonspecific interruption of learning process or unknown compensatory changes contribute to the disrupted CPP performance. Indeed, the results obtained from the genetic studies would be more convincing if the specificity of the genetic effects could be sufficiently addressed in the experimental design.

Studies using genetically manipulated mice have yielded important data on the roles that particular substrates play on ethanol's rewarding effects. However, it is difficult to determine whether the observed changes in ethanol CPP are attributable to a given gene's role or unknown compensatory changes resulting from the gene deletion itself. Thus, pharmacological studies are a key supplemental approach to genetic manipulations in understanding the neurochemical mechanisms underlying ethanol CPP. The sections below are devoted to pharmacological studies on ethanol CPP.

### Dopamine

Dopamine is known to play a critical role in the rewarding effects of ethanol and other abused drugs. Ethanol causes the release of dopamine in the nucleus accumbens (NAc)[61,62,63] and increases the firing rate of dopaminergic neurons in the ventral tegmental area (VTA)[64,65]. Systemic and intra-NAc injection of dopamine receptor antagonists have also been consistently shown to reduce ethanol's reinforcing effects and, ultimately, ethanol consumption[66,67,68]. However, evidence for the role of the dopaminergic system in ethanol CPP is somewhat controversial. Dopamine $D_1$ (SCH 23990) and $D_2$ (sulpiride) receptor antagonists have been shown to inhibit the expression of ethanol CPP in rats exposed to conditioned fear stress[69]. In contrast, the expression of ethanol CPP remained intact in mice following blockade of $D_1$ (SCH23390) or $D_2$ (raclopride or haloperidol) receptors[70,71]. Although the discrepancy between these results might be attributable to species differences, they may also be caused by procedural differences (e.g., the use of the fear conditioning procedure). Notably, genetic studies have not found a significant correlation between ethanol preference and dopamine $D_2$ receptor binding or *Drd2* gene expression in mice[72]. Thus, a dopamine-independent mechanism underlying ethanol CPP, at least in mice, appears to be likely.

Little research has examined the effect of dopamine $D_3$ and $D_4$ receptors on ethanol's conditioned effects. To date, some studies have demonstrated enhanced acquisition, but not expression, of ethanol CPP induced by inhibition of $D_3$ receptors in mice[70,73]. A clozapine study showed that the $D_4$ receptor has a similar lack of effect on the rewarding and reinforcing effects of ethanol[74]. Thus, despite the overwhelming evidence that dopamine systems, particularly the $D_2$ receptor, mediate ethanol's rewarding effects, most ethanol CPP studies in mice have failed to find evidence for this link.

Substantial evidence also implicates the mesolimbic dopamine pathway in the effects of ethanol. The cell bodies of the mesolimbic dopamine pathway originate in the VTA and project to several brain structures, mainly the NAc and prefrontal cortex[75]. Increased firing rates have been found in dopaminergic neurons located in the VTA projection to the NAc shell after ethanol administration[65,76]. Additionally, studies have recently determined the specific circuitry underlying the expression of ethanol CPP. Walker and Ettenberg[77] reported that intra-NAc shell dopamine receptor blockade suppressed both the development and expression of CPP induced by intracerebroventricular (i.c.v.) infusions of ethanol. Hill et al. investigated the distribution of Fos immunoreactivity in the brain via immunohistochemistry during ethanol CPP and conditioned place aversion in mice that received unpaired ethanol injections in their home cage or saline only (controls)[78]. Compared with control, c-*fos* expression increased only in the ethanol CPP group, predominantly in the mesolimbic dopamine pathway, extended amygdala, and hippocampus[78]. In contrast, such increased regional activity was not evident in animals that exhibited a conditioned place aversion. Thus, converging evidence strongly implicates dopamine in the acquisition and expression of ethanol CPP.

### Glutamate

Ethanol CPP research has traditionally focused on the dopaminergic system, but recent preclinical and clinical studies show that glutamate receptors also play a key role in the rewarding effects of ethanol[79,80]. Of the various ionotropic and metabotropic glutamate receptors, the receptor class most affected by ethanol is NMDA, an ion channel that causes localized depolarization when activated. Studies on the role of NMDA receptors in ethanol CPP have generated conflicting findings. For example, blockade of NMDA receptors by noncompetitive NMDA receptor antagonists (e.g., neramexane and MK-801) abolished both the acquisition and expression of ethanol CPP in rats[81,82]. The specific role of different NMDA receptor subunits in ethanol CPP has been further investigated in mice. Blockade of NMDA receptor glutamate binding sites by CGP-37849 blocked both ethanol CPP and conditioned place aversion in mice[83]. However, CGP-37849 also blocked the acquisition of LiCl-induced conditioned place aversion, raising the possibility that this inhibitory effect may be caused by a nonspecific alteration of learning ability[83]. Furthermore, inhibition of the strychnine-insensitive glycine site of the NMDA receptor complex prevented acquisition of ethanol CPP in rats[82], while administration of the glycine$_B$ partial agonist (+)-HA-966 and the NMDA NR2B receptor subunit antagonists fluphenazine and ifenprodil had no effect in mice[59,83].

The possibility exists that the seemingly contrasting effects of NMDA receptor antagonism on ethanol CPP and ethanol's other behavioral consequences may reflect the unique neurochemical and molecular mechanisms underlying the processes that mediate different ethanol-reinforced behaviors. Notably, however, species differences may also account for the conflicting findings regarding the effect of NMDA receptor glycine binding sites on ethanol CPP.

Despite the large body of evidence implicating NMDA receptors in the effects of ethanol, few studies have addressed the other glutamate receptor subtypes. We found only one study that examined the effect of the selective metabotropic glutamate receptor 5 (mGluR5) antagonist 2-methyl-6-(phenylethynyl)-pyridine (MPEP), which failed to alter the development of ethanol CPP[84]. Ethanol has also been shown to inhibit synaptic currents mediated by non-NMDA receptors and to inhibit presynaptic glutamate release via *N*-type calcium channels in the central nucleus of the amygdala[85]. In summary, despite many studies on the involvement of glutamate receptors in ethanol CPP, a clear idea of the role different glutamate receptors play in ethanol CPP has yet to emerge.

### *Serotonin*

Serotonin may modulate activity of the dopamine system. Binding studies have shown high to moderate densities of 5-HT receptors in the mesolimbic dopamine pathway, such as the NAc, that receives serotonergic projections from the dorsal raphe nucleus[86,87,88]. Ethanol-stimulated release of serotonin and dopamine in the NAc occurred via 5-$HT_3$ receptor activation and was blocked by 5-$HT_3$ receptor antagonists[89,90,91]. Several serotonin receptor subtypes (5-$HT_{1A}$, 5-$HT_{1B}$, 5-$HT_2$, and 5-$HT_3$) have been implicated in various ethanol-related behaviors, such as alcohol consumption, intoxication, withdrawal, and reward[92]. A number of studies have assessed the role of serotonin receptor subtypes in ethanol CPP in both mice and rats. For example, blockade of 5-$HT_{1A}$ receptors[93] or inhibition of serotonin uptake[94] did not alter the establishment of ethanol CPP, while inhibition of 5-$HT_2$ receptors enhanced the acquisition of ethanol CPP in mice[95]. In contrast, pretreatment with the selective 5-$HT_3$ receptor antagonist ondansetron attenuated the expression of ethanol CPP in rats exposed to conditioned fear stress[96]. However, in this latter study, intermittent electric footshocks were introduced prior to conditioning testing, and it is unclear whether the changes in the conditioning responses were mainly attributable to the effect of ethanol or the history of fear conditioning. Provided the complex contribution of 5-HT receptor subtypes to ethanol CPP, the possibility exists that different subtypes have differential, even opposite, effects on ethanol CPP.

### *Opioids*

A small number of studies have addressed the potential contribution of the endogenous opioid system to the rewarding effects of ethanol. Coadministration of morphine or heroin can potentiate the ability of low-dose ethanol to induce CPP in rats[97] and mice[77]. Naloxone, a nonselective opiate antagonist, failed to alter the acquisition and expression of ethanol CPP in both mice[98] and rats[99], but attenuated the potentiation of ethanol CPP acquisition induced by conditioned fear stress in rats[100]. Despite the lack of effect of nonselective opioid antagonists, the availability of highly selective opioid receptor antagonists has allowed the investigation of the specific roles that different opioid receptor subtypes play in ethanol CPP. For example, blockade of $\mu$ and $\delta$ receptors prevented the establishment of ethanol CPP, while inhibition of $\kappa$ receptors potentiated ethanol CPP in rats[100]. Stimulation of opioid receptor-like receptor 1 has been shown to reduce the acquisition and expression of ethanol CPP in mice[98]. Furthermore, intra-VTA injections of the nonselective opioid antagonist methylnaloxonium prevented the acquisition of ethanol CPP, whereas administration directly into the NAc had no effect in mice[101]. These studies show a critical, but also differential, role of opioid receptors in ethanol CPP.

The hypothesis that opioid receptors play a significant role in ethanol CPP is challenged by the finding that i.c.v. injections of the antiopioid peptide neuropeptide FF (NPFF) or the NPFF agonist 1DMe failed to suppress the expression of ethanol CPP in rats[102] and mice[103]. These studies share the potential limitation that the peptide's effectiveness in preventing endogenous opioid action in reward-related areas, such as the VTA after ventricular injection, has not been demonstrated. The discrepancy between these results might also be attributable to species differences. However, the majority of these studies suggest that the endogenous opioid system can greatly influence the induction and expression of ethanol CPP.

### *γ-Aminobutyric Acid*

γ-Aminobutyric acid (GABA) is the brain's major inhibitory neurotransmitter. It acts primarily through $GABA_A$, $GABA_B$, and $GABA_c$ receptors. Ethanol appears to interact directly with the GABA receptor complex and allosterically alters GABA function in specific brain regions[104]. Activation of $GABA_A$ receptors increases the acquisition of ethanol CPP[105], while intra-VTA infusion of baclofen, a $GABA_B$ agonist, reduces the establishment of ethanol CPP in male DBA/2J mice[101]. Notably, however, neither systemic nor intra-NAc injections of baclofen altered the magnitude of ethanol CPP, suggesting that the role of $GABA_B$ receptors on ethanol CPP is likely brain region specific[101]. These findings suggest that the GABAergic system plays an active role in ethanol CPP.

#### *Others*

The cannabinoid system comprises both cannabinoid $CB_1$ and $CB_2$ receptors. $CB_1$ receptors are predominantly localized in the brain and may modulate brain reward pathways in a dopamine-dependent fashion[106,107,108]. To date, no pharmacological studies have investigated the effect of $CB_1$ blockade on ethanol CPP. However, two genetic studies have demonstrated reduced ethanol CPP in $CB_1$ knockout mice, an effect that correlated with overexpression of striatal dopamine $D_2$ receptors[50,109], suggesting that $CB_1$ receptors play a key role in the development of dopamine-dependent ethanol CPP.

Ethanol CPP is also likely influenced by neuronal substrates other than the primary neurotransmitters described above. For example, the taurine derivative acamprosate dose dependently reduced the development of ethanol CPP, possibly by antagonizing glutamate NMDA receptors[110,111,112]. Similarly, inactivation of the primary ethanol metabolite by D-penicillamine also blocked the acquisition of ethanol CPP; whereas, the expression of ethanol CPP remained intact[113]. Pretreatment with methylenedioxymethamphetamine (MDMA; a serotonergic neurotoxin) prevented the formation of ethanol CPP in adult male Lister hooded rats[114]. Similarly, Itzhak and Martin[115] reported that the neuronal nitric oxide inhibitor 7-nitroindazole (7-NI) completely blocked ethanol CPP. In contrast, neither activation of benzodiazepine receptors[116] nor blockade of calcium channels[117] affected the acquisition of ethanol CPP.

### Extinction and Reinstatement of Ethanol CPP

Similar to cocaine and heroin CPP, previously established ethanol CPP can be extinguished by repeated exposure to the drug-paired environment in a drug-free state. This extinguished response can then be reinstated with a priming injection of low-dose ethanol[98,118,119]. This extinction procedure also eliminates ethanol conditioned place aversion[119]. Notably, these studies used mice because of their greater sensitivity to ethanol CPP than rats[38]. To date, few pharmacological studies have addressed the extinction and reinstatement of ethanol CPP, and those that exist have focused on the opioid system. Naloxone appears to have differential effects on the extinction and reinstatement of ethanol CPP. It facilitated extinction in DBA/2 J mice[118] and inhibited the reinstatement of ethanol CPP in NMRI mice[98]. The finding that naloxone influences the maintenance, extinction, and reinstatement of ethanol CPP suggests that endogenous opioids play a role in all phases of ethanol CPP.

## NICOTINE CONDITIONED PLACE PREFERENCE

### Establishment of Nicotine CPP

Many studies have revealed the difficulty in obtaining reliable nicotine CPP[see 120]. An analysis of 19 studies of nicotine CPP in rats published between 1985 and 2004 found that six failed to obtain nicotine CPP, nine reported nicotine CPP, and four reported nicotine conditioned place aversion[120]. Similar to ethanol, experimental conditions have great importance for creating nicotine CPP. For example, nicotine CPP can be facilitated by decreasing the animals' stress (e.g., by prolonged handling before conditioning), using the optimal nicotine dose (between 0.1 and 1 mg/kg), using brief conditioning sessions (20 min), using a biased procedure (i.e., pairing the CS with drug based on the animal's initial response), and using a species that is susceptible to nicotine CPP[120,121,122]. The animal's age at first nicotine administration may also be important. Increased nicotine CPP was evident in adolescent rats and adult rats that were previously exposed to nicotine[123,124,125,126]. By modifying the nicotine CPP methodology according to these findings, progressively more laboratories have produced significant nicotine-induced CPP in rats[120,127,128,129,130] and mice[131,132,133].

Evidence is also accumulating that nicotinic receptors are functionally important on dopaminergic neurons[134,135]. For example, nicotine-stimulated dopamine release in the ventral striatum was absent in β2 subunit nicotine receptor mutant mice[136,137], but could be restored by re-expressing the β2 subunit of the nicotinic acetylcholine receptor (nAChR) in β2 mutant mice[138]. Additionally, a substantial reduction of dopamine release in striatal synaptosomes was found only in α4 knockout, but not in α6 knockout mice[134]. The association between nicotinic receptors and dopaminergic neurons has been fairly well documented in the literature, but a growing number of reports has explored the influence of other neurotransmitter systems and neuronal substrates. Reduced nicotine CPP was observed in mice with genetic deletion of preproenkephalin[139] and μ opioid receptors[140]. The neurobiological mechanisms underlying nicotine's effects are still poorly understood and these genetic studies provide some useful insights into the possible effect of specific neuronal processes involved in nicotine CPP.

### Dopamine

Ample evidence has demonstrated abundant expression of nicotinic acetylcholine receptors in dopaminergic soma and cell bodies[141,142,143]. Increased dopamine release in the NAc is caused by systemic administration[144], intra-VTA infusion[145], or a systemic challenge of nicotine following chronic nicotine administration[146]. In contrast, a substantial reduction of dopamine release was observed in nicotine self-administering animals during early abstinence from chronic exposure[147]. Deactivation of the VTA by nicotinic antagonists or direct blockade of dopamine receptors have been shown to reduce nicotine self-administration significantly[148,149,150,151]. Altogether, these findings provide solid support for the hypothesis that the dopaminergic system plays a critical role in nicotine's behavioral effects. Indeed, systemic injection[152] or intra-NAc shell infusion of $D_1$ receptor antagonists[128] have been shown to block nicotine CPP. However, the latter study also found that blockade of $D_2$ receptors did not effectively alter nicotine CPP. Selective inhibition of $D_3$ receptors dose dependently attenuated nicotine CPP[153,154].

These findings with dopamine $D_3$ receptors are particularly intriguing because $D_3$ receptors may be critically involved both in the reinstatement of previously extinguished drug-seeking behaviors under operant conditioning and the response to drug-associated stimuli under Pavlovian conditioning[153,155,156]. Most likely, the inhibitory role of $D_3$ receptors in nicotine's conditioned effects may reflect the drug's disruptive effects on the influence of environmental stimuli on drug seeking[157,158].

### Glutamate

Nicotine stimulates the release of synaptic glutamate currents in various brain regions[159,160] and the simultaneous increases in dopamine levels can be reversed by an excitatory amino acid antagonist[161]. Consistent with these findings, a dose of nicotine that produced CPP also enhanced the concentrations of brain amino acids, including glutamate, in the NAc[162]. Some research has suggested that the action of glutamate via NMDA-sensitive receptors within the VTA is particularly important for nicotine CPP[160,163,164]. Indeed, administration of glutamate antagonists, such as ACPC (1-aminocyclopropanecarboxylic acid, a partial agonist at the strychnine-insensitive glycine receptor site on the NMDA receptor complex and a functional NMDA antagonist) blocked the acquisition of nicotine CPP[165]. In contrast, blockade of mGluR5 receptors had no effect on the formation of nicotine CPP[84]. These findings suggest that nicotine CPP appears to be mediated by glutamate receptors, especially NMDA receptors.

#### *Serotonin*

Serotonergic terminals make synaptic connections with both dopaminergic cell bodies and terminals[166,167], and dopaminergic system function has been hypothesized to be partially mediated by serotonin. As mentioned above, a key effect of nicotine is up-regulation of dopaminergic function. Thus, activation or deactivation of serotonin receptors likely produces dopamine-dependent changes in nicotine CPP, despite the fact that nicotine-stimulated release of serotonin is also evident in the cingulate frontal cortex[161]. Carboni et al.[168] reported that selective 5-$HT_3$ blockade prevented nicotine CPP and nicotine-stimulated dopamine release in the NAc[169]. A recent study reported that both stimulation of 5-$HT_{2C}$ and disruption of PTEN (phosphatase and tensin homolog deleted on chromosome 10) coupling with 5-$HT_{2C}$ receptors had a profound influence on nicotine CPP[170]. This disruption was also attributable to tonic alteration of the firing rate of dopaminergic neurons in the VTA[170,171,172].

#### *Opioids*

Nicotine's ability to induce the release of endogenous opioids in the brain[173,174,175] has prompted investigations of the role that the opioid system plays in nicotine CPP. Nonselective inhibition of opioid receptors by dipeptide synthesis inhibitor, glycyl-glutamine, blocked the acquisition and expression of nicotine CPP[176]. More specifically, $\mu$ receptor inhibition blocked both the acquisition[177] and expression[140] of nicotine CPP. Furthermore, enhanced cyclic adenosine monophosphate (cAMP) response-element binding protein (CREB) phosphorylation by exposure to the nicotine-paired environment was reversed by naloxone[140]. These findings suggest that $\mu$ opioid receptor activation is necessary for nicotine CPP.

#### *γ-Aminobutyric Acid*

The GABAergic system has been implicated in the development of addiction to various abused drugs, including nicotine, because of the direct antagonism that occurs between GABAergic and dopaminergic systems in the brain[178,179,180]. Nicotine CPP may be associated with increased dopamine release under inhibitory control of the GABAergic system. Indeed, inhibition of GABA transaminase by GVG (γ-vinyl GABA) or ACC (1*R*,4*S*-4-amino-cyclopent-2-ene-carboxylic acid) prevented both the acquisition and expression of nicotine CPP[181,182]. Moreover, GVG reduced extracellular dopamine levels in the NAc, suggesting that the inhibitory effect of GABA on dopamine release may be the primary cause of the reduced ethanol CPP response[182].

Although increased GABA levels, which occur following suppression of GABA transaminase, nonselectively interact with all subtypes of GABA receptors, the fact that attenuation of dopamine release can be reversed by a $GABA_B$ receptor agonist suggests that the $GABA_B$ subtype might be the principle determinant for the precise role of GABA in nicotine CPP. The $GABA_B$ receptor-positive modulator GS39783 blocked the acquisition of nicotine CPP[183]. More importantly, this study also found a significant correlation between ΔFosB induction and nicotine CPP, suggesting that this molecular adaptation occurs during the induction of nicotine CPP. In contrast, the expression of nicotine-induced CPP and ΔFosB remained intact when GS39783 was administered immediately before the postconditioning phase. The lack of effect of GS39783 on nicotine CPP may be attributable to the $GABA_B$ receptor-positive modulator's inability to affect nicotine CPP[183]. These findings provide a rationale for conducting more systematic studies of the effects of GABA on nicotine CPP to improve our understanding of the neurochemical mechanisms underlying this process.

#### *Cannabinoids*

The finding that nicotine did not induce CPP in $CB_1$ receptor–deficient mice suggests that cannabinoids play a role in nicotine CPP[184]. This hypothesis is supported by the finding that both the development and expression of nicotine CPP can be inhibited by acute injections of the $CB_1$ receptor antagonist rimonabant[127,185]. Interestingly, rimonabant more strongly inhibited the expression of nicotine CPP when it was administered 24 h after the last conditioning session, rather than 3 or 12 weeks after conditioning[127]. A study of the time course of $CB_1$ blockade further confirmed the presence of $CB_1$-dependent and -independent expression of nicotine CPP[186]. These data suggest that $CB_1$ receptors play a temporal role in nicotine CPP. A $CB_1$ receptor–dependent process may occur immediately after CPP induction, and a $CB_1$ receptor–independent effect may occur at later time points.

#### *Others*

A relative paucity of pharmacological studies exist that have assessed the roles that other neuronal substrates play in nicotine CPP. However, several genetic studies have revealed a complex interaction of the influences of multiple neuronal substrates in nicotine CPP. For example, mice with genetic deletion of adenosine A2a receptors[187] exhibited reduced nicotine-induced CPP. Moreover, CREBαΔ mutant mice failed to exhibit CPP for low-dose nicotine (1.0 mg/kg), and the place aversion the animals exhibited for 2.0 mg/kg nicotine remained unaltered compared with wild-type controls[140]. Deletion of ΔFosB has also been shown to reduce nicotine CPP[188].

#### Extinction and Reinstatement of Nicotine CPP

To the best of our knowledge, only one study has assessed extinction and reinstatement of nicotine CPP. Similar to CPP for other drugs, previously acquired nicotine CPP can be extinguished by allowing rats to explore both CPP apparatus chambers without any drug injections for several daily sessions[189]. In this study, both nicotine and morphine effectively reinstated extinguished CPP, and reinstatement was dose-dependently blocked by the calcium channel blockers nimodipine and flunarizine[189].

### CAFFEINE CONDITIONED PLACE PREFERENCE

Caffeine may be the most widely used psychoactive drug and its reinforcing effects in humans are well documented[190]. However, caffeine's rewarding effects have not been studied as extensively as those of cocaine and amphetamines in the CPP model. Caffeine was initially hypothesized to not induce CPP because rats did not exhibit significant CPP to flavor, cues, or flavor/cues paired with intraperitoneal injections of the drug[191]. However, Brockwell et al.[192] demonstrated that caffeine could induce significant CPP and found that caffeine produced a biphasic conditioning effect in which a lower dose was rewarding, but a higher dose was aversive. Two subsequent studies not only confirmed the biphasic nature of caffeine CPP, but also directly compared nicotine CPP with CPP for other drugs[193,194]. They discovered that caffeine is a weaker reinforcer than cocaine with regard to its conditioning ability[193] and that a low-dose combination of the two drugs can synergistically affect CPP[194].

To the best of our knowledge, no pharmacological studies have directly examined caffeine CPP. However, similar receptor antagonists have been studied. Caffeine is a nonselective adenosine antagonist, and the conditioning effects of the selective adenosine A1 receptor antagonist CGS 15943A and the A2

receptor antagonist CPX have been examined in the CPP procedure to determine their relative contribution to caffeine CPP[195]. Adenosine A2 receptor subtypes appear to play a more important role in the establishment of CPP than A1 receptor subtypes. Robust caffeine CPP was evident following administration of A2, but not A1, receptor antagonists[195]. To our knowledge, no studies have focused on the extinction and reinstatement of caffeine CPP, and further studies are certainly necessary to explore the behavioral, pharmacological, and molecular mechanisms of caffeine CPP.

### CONCLUDING REMARKS

The CPP model is a widely accepted behavioral model used to explore the neural and behavioral mechanisms underlying the rewarding effects of abused drugs[5,20]. This model has enabled investigators to study the conditioned and rewarding properties of the world's most commonly used drugs: ethanol, nicotine, and caffeine. Through this research, various neuronal substrates have been found to contribute to ethanol- and nicotine-induced CPP (Table 1). Ethanol and nicotine, like other abused drugs, activate multiple neurotransmitter systems. Using the CPP paradigm, a large body of research has provided strong evidence that both ethanol and nicotine depend on dopamine for their rewarding effects. Additionally, dopamine appears to play an important role in all phases of ethanol and nicotine CPP, including acquisition, expression, extinction, and reinstatement. In contrast, the effects of other neurotransmitters on ethanol/nicotine CPP are largely determined by the subtypes of their respective receptors. Thus, determination of the selective pharmacological blockade of ethanol/nicotine CPP during various phases is needed for a more complete understanding of the specific role of different neurotransmitters in the rewarding effects of ethanol and nicotine. To date, no pharmacological studies have directly examined caffeine CPP and further studies are certainly in great need to explore the neurochemical mechanism underlying the rewarding effect of caffeine.

While these findings have significantly increased our understanding of these commonly used drugs, we believe that no single behavioral procedure is sufficient to explore these drugs' complex addictive properties fully. Instead, the CPP procedure should be used specifically to examine these drugs' rewarding effects. The relative simplicity of the CPP paradigm makes it an excellent tool to better understand the behavioral and neurochemical attributes of these drugs' conditioned effects and to investigate the modulation of the rewarding effects of ethanol, nicotine, and caffeine.

### ACKNOWLEDGMENTS

This work was supported in part by a grant from the National Basic Research Program of China (973 Program, 2007CB512302) (LL), the National High Technology Research and Development Program of China (863 Program, 2006AA02Z4D1) (LL), the Natural Science Foundation of China (No: 30570576 and 30670713) (LL), and a CIHR-TUSP new investigator award (BLF). We thank Dr. Yavin Shaham for his critical comments on this manuscript.

**TABLE 1**
**Establishment of Drug-Induced CPP**

| Major Neurotransmitter | Drug | Major Findings | Selected References |
|---|---|---|---|
| Dopamine | *Ethanol* | Blocked by $D_1$, $D_2$, and $D_3$ (only acquisition) antagonist; $D_4$ no effect | 69,70,71,72,73,74 |
| | *Nicotine* | Blocked by $D_1$ and $D_3$ antagonists, $D_3$ partial agonist; $D_2$ no effect | 128,153,154 |
| Glutamate | *Ethanol* | Conflicting results of NMDA antagonist; mGlu5 antagonist no effect | 81,82,83,84 |
| | *Nicotine* | Blocked by NMDA antagonists and partial NMDA agonist; mGlu5 no effect | 165,168,169,170 |
| Serotonin | *Ethanol* | Acquisition potentiated by 5-$HT_2$ antagonist; blocked by 5-$HT_3$ antagonist; 5-$HT_{1A}$ antagonist and selective serotonin reuptake inhibitor no effect | 93,94,95,96 |
| | *Nicotine* | Blocked by 5-$HT_3$ antagonists; potentiated by 5-$HT_{2C}$ antagonist | 84,165 |
| Opioid | *Ethanol* | Potentiated by morphine or heroin; $\kappa$ antagonist; blocked by $\mu$ and $\delta$ antagonists and opioid-like receptor 1 antagonist | 77,97,98,99,100,101 |
| | *Nicotine* | Blocked by nonselective opioid antagonist and dipeptide synthesis inhibitor | 168,169,170 |
| GABA | *Ethanol* | Potentiated by $GABA_A$ agonist; blocked by $GABA_B$ agonist | 101,105 |
| | *Nicotine* | Blocked by GABA transaminase inhibitor, $GABA_B$ positive modulators (only acquisition) | 181,182 |
| Cannabinoid | *Ethanol* | Not tested | Not tested |
| | *Nicotine* | Blocked by $CB_1$ antagonist (short- and long-term expression was differentially affected) | 127,185,186 |
